# Advanced Photocatalysts for CO_2_ Conversion by Severe Plastic Deformation (SPD)

**DOI:** 10.3390/ma16031081

**Published:** 2023-01-26

**Authors:** Saeid Akrami, Tatsumi Ishihara, Masayoshi Fuji, Kaveh Edalati

**Affiliations:** 1 Department of Life Science and Applied Chemistry, Nagoya Institute of Technology, Tajimi 507-0071, Japan; 2 WPI International Institute for Carbon-Neutral Energy Research (WPI-I2CNER), Kyushu University, Fukuoka 819-0395, Japan; 3 Mitsui Chemicals, Inc.—Carbon Neutral Research Center (MCI-CNRC), Kyushu University, Fukuoka 819-0395, Japan; 4 Department of Applied Chemistry, Faculty of Engineering, Kyushu University, Fukuoka 819-0395, Japan; 5 Advanced Ceramics Research Center, Nagoya Institute of Technology, Tajimi 507-0071, Japan

**Keywords:** functional properties, ultrafine-grained (UFG) materials, nanostructured materials, photocatalytic CO_2_ conversion, high-pressure torsion (HPT), oxygen vacancies, high-pressure phases, high-entropy ceramics

## Abstract

Excessive CO_2_ emission from fossil fuel usage has resulted in global warming and environmental crises. To solve this problem, the photocatalytic conversion of CO_2_ to CO or useful components is a new strategy that has received significant attention. The main challenge in this regard is exploring photocatalysts with high efficiency for CO_2_ photoreduction. Severe plastic deformation (SPD) through the high-pressure torsion (HPT) process has been effectively used in recent years to develop novel active catalysts for CO_2_ conversion. These active photocatalysts have been designed based on four main strategies: (i) oxygen vacancy and strain engineering, (ii) stabilization of high-pressure phases, (iii) synthesis of defective high-entropy oxides, and (iv) synthesis of low-bandgap high-entropy oxynitrides. These strategies can enhance the photocatalytic efficiency compared with conventional and benchmark photocatalysts by improving CO_2_ adsorption, increasing light absorbance, aligning the band structure, narrowing the bandgap, accelerating the charge carrier migration, suppressing the recombination rate of electrons and holes, and providing active sites for photocatalytic reactions. This article reviews recent progress in the application of SPD to develop functional ceramics for photocatalytic CO_2_ conversion.

## 1. Introduction

**Figure 1:**
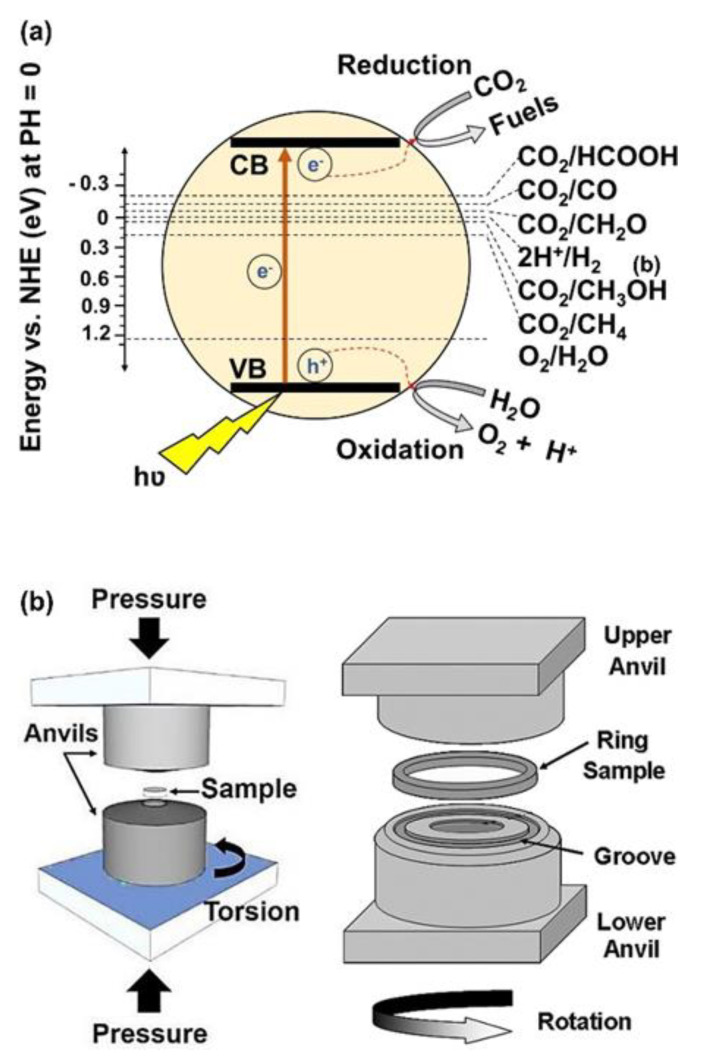
Schematic illustration of (**a**) photocatalytic CO_2_ conversion and (**b**) high-pressure torsion [41].

Nowadays, environmental crises, especially global warming caused by CO_2_ emission from burning fossil fuels and humankind activities, are considered one of the most significant challenges in the world. Reduction of CO_2_ to reactive CO gas or useful components and fuels, such as CH_4_ and CH_3_OH, using photocatalysts is one of the clean and new strategies, which is developing rapidly [1,2,3]. In photocatalytic CO_2_ conversion, excited electrons transfer from the valence band to the conduction band of the photocatalysts by solar irradiation and contribute to the reduction of CO_2_ to form desirable products, as shown in Figure 1a [3]. To perform these reduction reactions, some thermodynamic and kinetic conditions should be provided. From the viewpoint of thermodynamics, the standard potential of the reduction and oxidation reactions should be between the valence band and the conduction band of the photocatalyst [3,4]. On the other hand, from the kinetic viewpoint, the electrons should absorb the light, transfer to the conduction band, migrate to the surface of the photocatalyst, and take part in the reactions before combining with the holes [3,4]. To satisfy these kinetic and thermodynamic conditions, a photocatalyst should have some features, including high light absorbance, appropriate band structure, low recombination rate of electrons and holes, easy migration of charge carriers, and high surface affinity to adsorb CO_2_ with abundant active sites [3,4]. A combination of these thermodynamic and kinetic factors determines the speed of the reactions and the type of final products in photocatalysis.

Semiconductors, such as TiO_2_ [5,6,7], g-C_3_N_4_ [8,9], and BiVO_4_ [10,11,12], are typical photocatalysts that have been engineered by various strategies to enhance the catalytic efficiency for CO_2_ conversion. Doping with impurities, such as nitrogen, phosphorous, copper, and palladium [13,14,15]; defect engineering [16,17]; strain engineering [18,19]; mesoporous structure production [20]; and heterojunction introduction [21,22] are some of the most promising strategies that have been used so far to improve the optical properties and catalytic activity of various photocatalysts. Among these strategies, doping with impurities is the most investigated and feasible method, but impurities can increase the recombination rate of electrons and holes [13,14,15]. Therefore, finding new strategies to improve the photocatalytic activity and suppress the recombination rate of electrons and holes is a key issue. In this regard, severe plastic deformation (SPD) through the high-pressure torsion (HPT) method, which is mainly used for nanostructuring of metallic materials, has been used as a new tool to develop active photocatalysts for water splitting [23,24,25,26,27,28,29,30], dye degradation [31,32,33,34], and especially CO_2_ conversion [35,36,37,38]. This method not only does not increase the recombination rate of electrons and holes but also effectively suppresses it and improves some other optical properties. The SPD method has also been used effectively to synthesize new families of catalysts, such as high-pressure photocatalysts and high-entropy photocatalysts [23,27].

This article reviews recent publications on the impact of SPD through the HPT method on photocatalytic activity for CO_2_ conversion. The four main strategies used for this purpose are discussed in detail: (i) oxygen vacancy and strain engineering, (ii) stabilization of high-pressure phases, (iii) synthesis of defective high-entropy oxides, and (iv) synthesis of low-bandgap high-entropy oxynitrides.

## 2. Influence of HPT on Photocatalytic CO_2_ Conversion

HPT as an SPD method has been used since 1935 until now for grain refinement and the production of nanostructured materials. In addition to grain refinement, the introduction of various defects, such as vacancies and dislocations, is another feature of HPT, which resulta in the improvement of the functionality of materials proceeded by this method [39,40]. In the HPT method, both large shear strain and high pressure (in the range of several gigapascals) are simultaneously utilized to process or synthesize various ranges of materials [39,40]. Strain and pressure are applied to the material (disc or ring shape) using two anvils that rotate with respect to each other, as shown in Figure 1b [41]. Due to the high processing pressure in HPT, it is applicable to hard and less ductile materials, such as high-melting temperature metals (hafnium [42], molybdenum [43], and tungsten [44]), amorphous glasses [45,46], silicon-based semiconductors [47,48], and even ultrahard diamond [49,50]. Another advantage of HPT is its capacity to induce ultra-SPD (i.e., shear strains over 1000 for mechanical alloying) [51]. The inducing ultra-SPD [51] together with fast dynamic diffusion [52,53] introduces the HPT method as a unique path to mechanically synthesize new materials even from immiscible systems [54,55]. Due to these unique features of HPT, the method was even used for the process and synthesis of hard and brittle ceramics, but the number of publications on ceramics is quite limited despite the high potential of these materials for various applications [23,24,25,26,27,28,29,30,31,32,33,34,35,36,37,38,56,57,58,59,60,61,62,63,64,65,66,67,68,69,70,71,72,73,74,75,76,77,78,79,80,81]. Published studies regarding ceramics processed or synthesized by HPT are presented in Table 1, although there are other classic publications on HPT processing of ceramics mainly by physicists and geologists [40].

As given in Table 1, the recent usage of HPT to process and synthesize ceramics for photocatalysis, especially photocatalytic CO_2_ conversion, has shown a high potential of this method for the enhancement of photocatalytic activity [35,36,37,38]. The HPT method effectively leads to increased efficiency by narrowing the bandgap, increasing the light absorbance, aligning the band structure, introducing the interphases and active sites for chemical adsorption and reaction, and accelerating the charge carrier migration [35,36,37,38]. While the HPT method can control all these features simultaneously by simple mechanical treatment, chemical methods are not usually able to improve all these features at the same time. The main drawbacks of the HPT method are the small quantity of the sample and the low specific surface area of the catalyst due to the high pressure and strain utilized. However, upscaling the HPT method and increasing the specific surface area by a post-HPT treatment are issues that can be addressed in the future. The improvement of features of photocatalysts by HPT has been achieved using four main strategies, including simultaneous strain and oxygen vacancy engineering, the introduction of high-pressure phases, the formation of defective high-entropy phases, and the production of low-bandgap high-entropy oxynitride phases. The responsibility of each mentioned strategy to improve the photocatalytic CO_2_ conversion activity is discussed in detail as follows. It should be noted that all photocatalytic CO_2_ conversion experiments on HPT-processed catalysts were performed in an aqueous liquid phase inside a quartz photoreactor with a continuous flow of CO into the liquid phase and NaHCO_3_ as the sacrificial agent.

### 2.1. Simultaneous Strain and Oxygen Vacancy Engineering

Oxygen vacancy engineering is an effective method that has been used to improve photocatalytic CO_2_ conversion. Oxygen vacancies increase the photocatalytic efficiency by increasing the light absorbance, accelerating the charge carrier separation, and enhancing the surface reactions [35,36]. Oxygen vacancies on the surface of the photocatalysts act as active sites to trap the electrons for various ranges of reduction reactions. It was also observed that oxygen vacancies have a significant role in adsorbing and activating the CO_2_ molecules and increasing the local electronic density [35,36].

**Figure 2:**
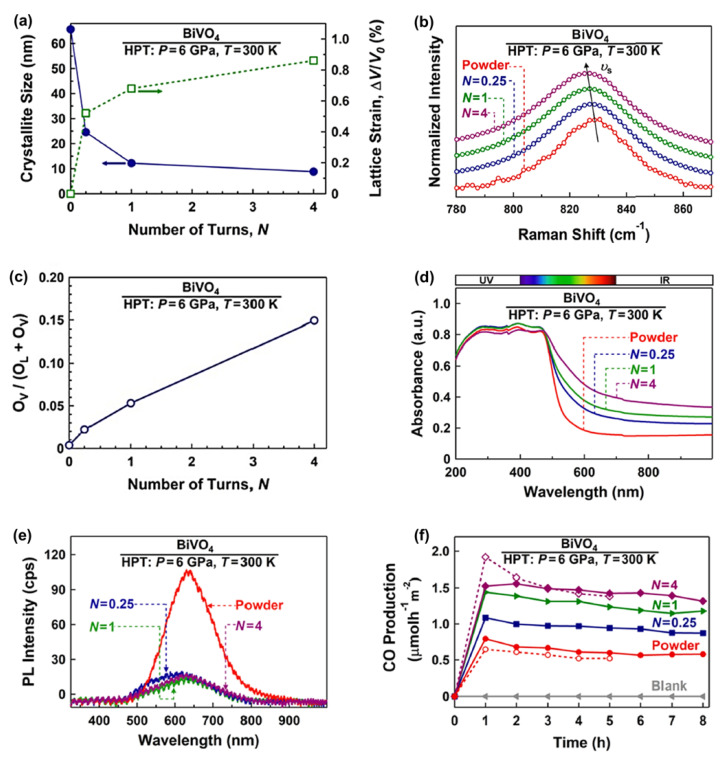
Improvement of light absorbance, suppression of recombination, and enhancement of photocatalytic CO_2_ conversion for BiVO_4_ by simultaneous strain and oxygen vacancy engineering using high-pressure torsion (HPT). (**a**) Crystallite size and volumetric strain versus the number of HPT turns (*N*), (**b**) Raman spectroscopy of initial and HPT-processed samples (inset: the appearance of samples), (**c**) oxygen vacancy concentration versus the number of HPT turns calculated by X-ray photoelectron spectroscopy, (**d**) UV–VIS spectroscopy, (**e**) photoluminescence spectra, and (**f**) photocatalytic CO production rate versus time for initial powder and sample proceeded by HPT for *N* = 0.25, 1, and 4 turns [35].

BiVO_4_ is one of the common photocatalysts utilized for photocatalytic CO_2_ conversion, but it suffers from a high recombination rate of electrons and holes and an inappropriate conduction band position [35]. Different strategies have been used to solve these problems, but in all of them, impurity atoms or a second phase are added to this material [35]. The HPT method was used to solve the problems of BiVO_4_ for photocatalytic CO_2_ conversion by simultaneous engineering of strain and oxygen vacancies without the addition of impurities. BiVO_4_ was processed by HPT for *N* = 0.25, 1, and 4 turns to investigate the impact of strain on photocatalytic properties and efficiency. Increasing the lattice strain and decreasing the crystallite size by increasing the HPT turns is shown in Figure 2a. The occurrence of lattice strain was also confirmed by Raman peak shift to lower wavenumbers, as shown in Figure 2b. It was also observed that the concentration of oxygen vacancies increases in BiVO_4_ by increasing the applied shear strain. Figure 2c illustrates the oxygen vacancy concentration, calculated by X-ray photoelectron spectroscopy (XPS), against the number of HPT turns, confirming that the concentration of vacancies increases by increasing the applied shear strain. Furthermore, strain and vacancy engineering led to an increase in light absorbance (Figure 2d) and a slight narrowing of the bandgap from 2.4 eV for the initial powder to 2.1 eV for the sample proceeded by HPT for *N* = 4 turns [35].

Simultaneous strain and oxygen vacancy engineering could significantly solve the problem of BiVO_4_ in terms of the high recombination rate of electrons and holes, as shown in Figure 2e. This figure demonstrates that the HPT method decreases the photoluminescence intensity, which is a piece of evidence for the suppression of recombination. Finally, this strategy was successful in improving the photocatalytic activity of BiVO_4_, as shown in Figure 2f. The CO production rate from CO_2_ photoreduction was effectively increased by increasing the number of HPT turns. This study was the first successful work that used simultaneous strain and oxygen vacancy engineering to improve the photocatalytic activity of BiVO_4_ without using impurities, suggesting SPD as a new path to improve the optical and electronic structure of photocatalysts for CO_2_ conversion [35].

### 2.2. Introducing High-Pressure Phases

**Figure 3:**
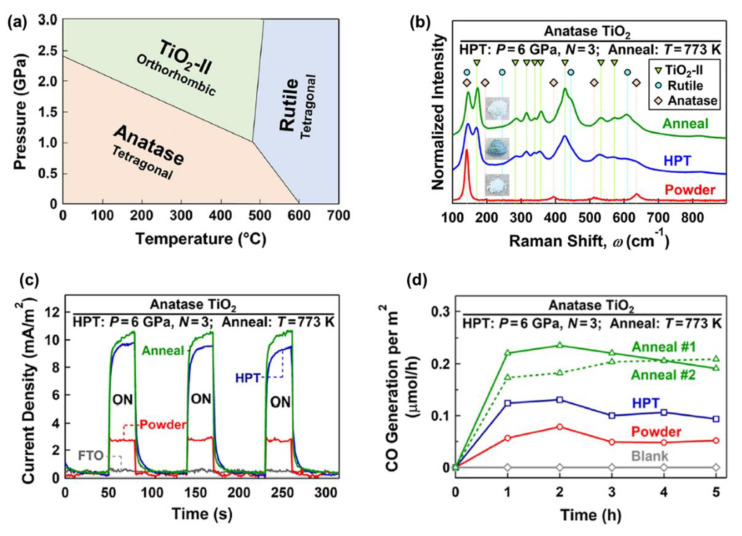
Improved charge carrier migration and photocatalytic CO_2_ conversion by introducing the high-pressure TiO_2_-II phase. (**a**) Pressure–temperature phase diagram of TiO_2_. (**b**) Raman spectra, (**c**) photocurrent spectra, and (**d**) photocatalytic CO production rate versus time for TiO_2_ before and after high-pressure torsion processing and after annealing [36].

The formation of high-pressure phases is one of the HPT effects that can occur for some ceramics, such as TiO_2_ [65], ZrO_2_ [58], ZnO [26], SiO_2_ [34], VO_2_ [78], Y_2_O_3_ [66], BaTiO_3_ [64], Al_2_O_3_ [27], and BN [68]. It was observed that these high-pressure phases contain defects, such as oxygen vacancies and dislocations, and have nanosized grains, which makes them attractive for photocatalytic applications. TiO_2_ with the anatase and rutile crystal structures is one of the most active photocatalysts for CO_2_ conversion. As shown in Figure 3a, in addition to anatase and rutile, TiO_2_ has a high-pressure TiO_2_-II (columbite) phase with the orthorhombic structure. Despite many studies on TiO_2_ photocatalysts, there was not any research work on photocatalytic CO_2_ conversion on the TiO_2_-II phase until 2021. Groups of current authors stabilized the TiO_2_-II phase by the HPT method and investigated it for photocatalytic CO_2_ conversion [36]. To decrease the fraction of oxygen vacancies in the bulk, which can act as recombination centers, an HPT-processed sample was further treated by annealing [36]. The formation of high-pressure TiO_2_-II was proved by X-ray diffraction (XRD), Raman spectroscopy, and transmission electron microscopy (TEM). Raman spectra along with the appearance of samples are shown in Figure 3b. New Raman peaks at wavenumbers 171, 283, 316, 340, 357, 428, 533, and 572 cm^−1^ correspond to the TiO_2_-II phase. The changes in the color of the sample from white to dark green after HPT processing and from dark green to white after annealing indicate that large fractions of oxygen vacancies are formed after HPT processing, while some of them are annihilated after annealing, a fact that was also proved by various characterization techniques [36].

The light absorbance of the TiO_2_-II phase produced by HPT processing was higher, and it had a narrower optical bandgap of 2.5 eV compared with anatase (3 eV), although the bandgap slightly increased to 2.7 eV after annealing [36]. Introducing the high-pressure TiO_2_-II phase using HPT suppressed the recombination rate of electrons and holes. It also had a positive impact on photocurrent generation, as shown in Figure 3c so that the annealed sample had the highest current density, suggesting the improvement of charge carrier separations by introducing the high-pressure TiO_2_-II phase. The potential of this new phase for CO_2_ adsorption was measured by attenuated total reflectance Fourier transform infrared (ATR-FTIR) spectroscopy. It was observed that the annealed sample had the highest potential for CO_2_ adsorption, which can help with photocatalytic CO_2_ conversion. Finally, this new phase showed a higher potential for photocatalytic CO production compared with the anatase phase, as shown in Figure 3d. The introduction of the TiO_2_-II phase with an optimized fraction of oxygen vacancies significantly improved the activity so that the annealed sample had the highest efficiency for CO_2_-to-CO conversion. The formation of anatase–columbite interphases can also contribute to the high activity of the HPT-processed sample by increasing the electron–hole separation and migration. In conclusion, high-pressure phases show great potential to be used as photocatalysts, and SPD can be used to stabilize these high-pressure phases under ambient conditions [36].

### 2.3. Formation of Defective High-Entropy Phases

Introducing high-entropy ceramics as new materials with five or more principal elements opened a new path in the field of materials science to produce materials with high functionality for various applications [81,82]. High configurational entropy caused by a large number of elements in these materials leads to decreasing the Gibbs free energy and improving the phase stability. High-entropy ceramics have been utilized for various applications, and in many cases, they have shown better efficiencies than conventional materials [81,82]. Li-ion batteries [83], catalysts [84], dielectrics [85], magnetic components [86], thermal barrier coating [87], and so on are some of the applications of these materials. The high potential of high-entropy ceramics for various applications is attributed to their high stability, cocktail effect, lattice distortion, inherent defects, and valence electron distribution [81,82]. Despite the high functionality of these materials, their application for photocatalytic CO_2_ conversion was not investigated until a study was conducted by the current authors in 2022 [37].

**Figure 4:**
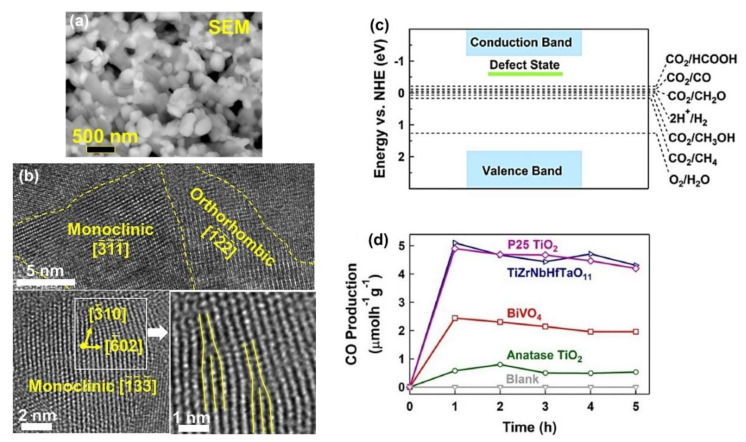
High photocatalytic CO_2_ conversion on defective high-entropy oxide TiZrNbHfTaO_11_ synthesized by high-pressure torsion. Microstructure of TiZrNbHfTaO_11_ by (**a**) scanning electron microscopy and (**b**) high-resolution transmission electron microscopy. (**c**) Electronic band structure of TiZrNbHfTaO_11_. (**d**) Photocatalytic CO production rate on TiZrNbHfTaO_11_ versus time compared with P25 TiO_2_, BiVO_4_, and anatase TiO_2_ [37].

The HPT method, followed by oxidation, was used to fabricate a defective high-entropy oxide (HEO) with the composition of TiZrNbHfTaO_11_ and dual crystal structure of monoclinic and orthorhombic [37]. The selection of elements for this high-entropy ceramic was conducted by considering the d^0^ electronic structure of cations that have shown high potential for photocatalysis. The oxidation states of anionic and cationic elements and their uniform distribution were proved by XPS and energy-dispersive X-ray spectroscopy (EDS), respectively. The microstructure of the oxide is shown in Figure 4a using scanning electron microscopy (SEM) and in Figure 4b using high-resolution TEM. In addition to a nanocrystalline dual-phase structure, the material exhibited the presence of various defects, such as vacancies and dislocations, as shown in Figure 4b. The formation of oxygen vacancies in this material was examined by electron paramagnetic resonance (EPR) spectroscopy. These oxygen vacancies can act as shallow traps between the valence band and the conduction band for easier charge carrier separation, as shown in Figure 4c [37].

TiZrNbHfTaO_11_ had a higher light absorbance and lower bandgap compared with the binary oxides, including TiO_2_, ZnO, Nb_2_O_5_, HfO_3_, and Ta_2_O_5_ [37]. This HEO could successfully generate photocurrent, which indicates its potential for easy separation of electrons and holes to improve photocatalytic activity. TiZrNbHfTaO_11_ showed higher activity for photocatalytic CO production compared with BiVO_4_ and TiO_2_ as two typical photocatalysts, as shown in Figure 4d. Additionally, it had the same photocatalytic activity as P25 TiO_2_ as a benchmark photocatalyst. The high activity of this HEO for photocatalytic CO_2_ conversion was attributed to the presence of defects, such as oxygen vacancies and dislocations; interphases; and high light absorbance. This study reported the first application of high-entropy ceramics for photocatalytic CO_2_ conversion and introduced a new way to design and synthesize highly efficient high-entropy photocatalysts by SPD processing [37].

### 2.4. Synthesis of Low-Bandgap High-Entropy Oxynitrides

Metal oxides are the most conventional photocatalysts for CO_2_ conversion but suffer from a large bandgap. On the other hand, metal nitrides have a low bandgap but suffer from low stability compared with metal oxides. Metal oxynitrides are rather new materials that can solve the problems of metal oxides and nitrides in terms of large bandgap and low stability, respectively [88]. Although oxynitrides have been used for photocatalytic water splitting in many research works, their application for photocatalytic CO_2_ conversion has been limitedly investigated mainly due to their limited chemical stability. The concept of high-entropy materials with high stability is one strategy used to produce high-entropy oxynitrides with low bandgap and high stability for CO_2_ photoreduction [38].

**Figure 5:**
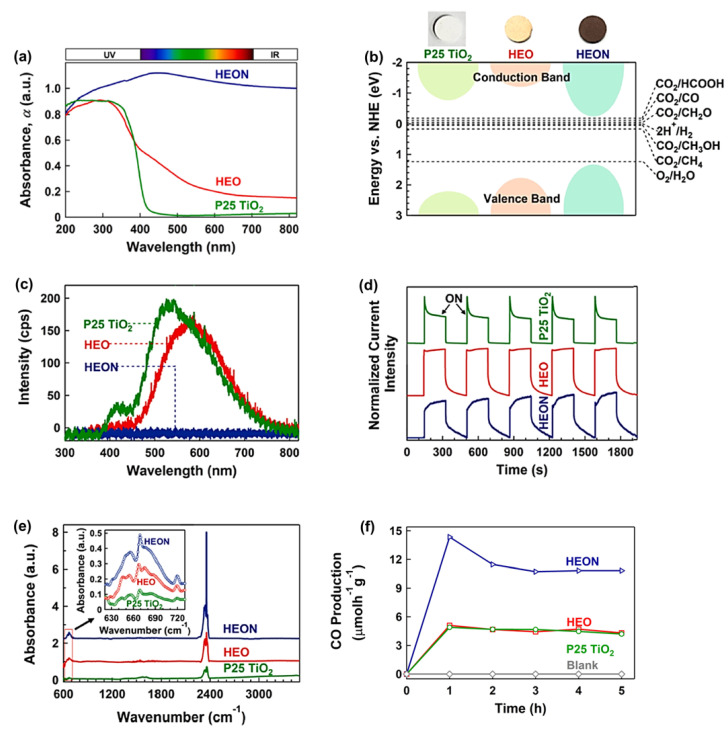
High light absorbance, appropriate band structure, suppressed recombination, significant CO_2_ adsorption, and high photocatalytic CO_2_ conversion for high-entropy oxynitride (HEON) TiZrNbHfTaO_6_N _3_. (**a**) UV–VIS light absorbance spectra, (**b**) electronic band structure together with the appearance of samples, (**c**) photoluminescence spectra, (**d**) photocurrent density versus time, (**e**) diffuse reflectance infrared Fourier transform spectra (peaks at 665 and 2350 cm^−1^ represent chemisorption and physisorption of CO_2_), and (**f**) photocatalytic CO production rate versus time for TiZrNbHfTaO_6_N_3_ compared with P25 TiO_2_ and high-entropy oxide (HEO) TiZrNbHfTaO_11_ [38].

A high-entropy oxynitride (HEON) with the composition of TiZrNbHfTaO_6_N_3_ was fabricated by the HPT method, followed by oxidation and nitriding, and its photocatalytic performance was compared with a corresponding HEO TiZrNbHfTaO_6_ and P25 TiO_2_ benchmark photocatalyst [38]. This HEON had dual phases with face-centered cubic (FCC) and monoclinic structures with uniform distribution of elements. This HEON material had much higher light absorbance compared with P25 TiO_2_ and relevant HEO, as shown in Figure 5a. It showed a superior low bandgap of 1.6 eV as one of the lowest bandgaps reported in the literature for oxynitride photocatalysts. The improved electronic band structure of this HEON compared with P25 TiO_2_ and HEO is shown in Figure 5b. The recombination rate of electrons and holes in HEON was low so that its photoluminescence intensity was negligible compared with P25 TiO_2_ and HEO (Figure 5c). The shape of photocurrent spectra shown in Figure 5d also confirmed the low recombination rate of electrons and holes in this HEON compared with the HEO and P25 TiO_2_ catalysts. The potential of this HEON for CO_2_ adsorption was measured by diffuse reflectance infrared Fourier transform (DRIFT) spectrometry, which showed the higher physical adsorption and chemisorption (in the form of carbonate) of CO_2_ on this HEON compared with P25 TiO_2_ and HEO (Figure 5e).

This HEON successfully converted CO_2_ to CO with extremely high efficiency even compared with the P25 TiO_2_ benchmark photocatalyst, as shown in Figure 5f. Although HEON could adsorb the light in both visible and infrared regions of light, it could not convert CO_2_ in these regions within the detection limits of the gas chromatograph. The stability of HEON was examined by conducting a long-term photocatalytic test for 20 h after storage of the sample in the air for 6 months. The photocatalytic activity of the material was not degraded, and X-ray diffraction analysis confirmed that the crystal structure of HEON did not change after 6-month storage and the long-time photocatalytic reaction. In conclusion, the low-bandgap HEON catalysts synthesized by SPD can be considered a new family of highly efficient photocatalysts for CO_2_ conversion [38].

## 3. Discussion on Future Outlook

The application of SPD to synthesize new photocatalysts for CO_2_ conversion introduced significant findings from the viewpoints of photocatalysis and SPD. The significance of these issues and their impact on the future outlook of this research field are discussed here.

For all these photocatalysts developed by HPT, CO was the only product that was detected using a flame ionization detector. The nonproduction of other products, such as CH_4_, CH_3_OH, HCOOH, or CH_2_=CH_2_, can be explained by considering the thermodynamic and kinetic parameters. For instance, CH_4_ is a product that thermodynamically is more feasible to be produced than CO due to its lower standard potential. However, more electrons are required to produce this component compared with CO [89]. Therefore, from the viewpoint of kinetics, CO production is more feasible than CH_4_ formation. Another point that should be considered is that CO has no tendency to be adsorbed to the active sites of the photocatalysts after production, which leads to propelling the reaction to CO production [89]. The production of CO as the only product can also be explained by the pathway of the reaction. In photocatalytic CO_2_ conversion, the formation of a ${\mathrm{CO}}_{2}^{•-}$ intermediate product is the initial step. This intermediate product is formed by interchanging the electrons between CO_2_ and the surface of the catalyst. Adsorption modes of ${\mathrm{CO}}_{2}^{•-}$ to the surface of the photocatalyst specify the reaction pathway. The ${\mathrm{CO}}_{2}^{•-}$ intermediate product can be adsorbed to the surface of the photocatalyst by three modes, which include (i) oxygen coordination, (ii) carbon coordination, and (iii) combination of oxygen and carbon coordination [90]. Oxygen coordination occurs when the photocatalyst is formed from tin, lead, mercury, indium, and cadmium metals. In this case, ^•^OCHO and formic acid are produced as intermediate and final products, respectively. If the noble and transition metals are the elements forming the photocatalyst, carbon coordination occurs and ^•^CO and CO are the intermediate and final products, respectively [90]. The presence of copper atoms in the structure of photocatalysts leads to the formation of a combination of oxygen and carbon coordination to produce both ^•^OCHO and ^•^CO as intermediates and CO, CH_4_, and C_2_H_5_OH as final products. Since all photocatalysts investigated by HPT include the transition metals, CO is the final product, and the reaction pathway can be considered as follows [90].

(1) $${\mathrm{CO}}_{2}+e^{-}\to {\mathrm{CO}}_{2}^{{•}^{-}}$$

(2) $${\mathrm{CO}}_{2}^{{•}^{-}}+2e^{-}+2H^{+}\to \mathrm{CO}+{\;H}_{2}O$$

Table 2 compares the photocatalytic activity of HEON synthesized by HPT with reported photocatalysts in the literature by normalizing the amount of CO production to catalyst mass and surface area [91,92,93,94,95,96,97,98,99,100,101,102,103,104,105,106,107,108,109,110,111,112,113,114,115,116,117,118,119,120,121,122,123]. Since the photocatalytic reaction occurs on the surface, comparing the results by normalizing them to the surface area is more reasonable. According to this table, the amount of CO production for HEON is 4.66 ± 0.3 µmolh^−1^m^−1^, which is higher than the best photocatalysts reported in the literature. This indicates that the contribution of SPD to introducing new families of photocatalysts will receive high appreciation in the future by considering the current demands in finding new strategies to deal with the CO_2_ emissions; however, the synthesis method and compositions are expected to be modified by the experts in the field of photocatalysis. For example, one main disadvantage of SPD for the process and synthesis of catalysts is the low surface area of the synthesized material, while large specific surface areas are desirable in catalysis [74]. Moreover, theoretical studies are required to clarify the mechanisms underlying the high activity of photocatalysts developed by SPD so that new catalysts can be designed.

The SPD field experienced significant progress in the past three decades, as discussed in several review papers [124,125,126,127,128,129], and more recently in a special issue in 2019 [130], which gathered overviews on both historical developments [131] and recent advancements [132]. A survey of these overviews indicates that despite significant progress on theoretical aspects [133,134], mechanisms [135,136], processing [137,138,139,140,141,142,143,144], microstructure [145,146,147,148,149], and mechanical properties [150,151,152,153,154,155] of metallic materials, there is a recent tendency to apply SPD to a wider range of materials (oxides [156], semiconductors [157], carbon polymorphs [158], glasses [159], and polymers [160]) to control phase transformations [161] and solid-state reaction [162,163,164] for achieving advanced functional properties [165,166,167,168,169,170,171,172]. CO_2_ conversion is perhaps the newest application of SPD to functional materials, which expanded the synthesis capability of SPD from metallic materials to ceramics [37,38]. Moreover, this application has led to the introduction of new benchmark photocatalysts, which can open new pathways and research directions in corresponding fields. Although the application of SPD for CO_2_ photoreduction is currently limited to the HPT method, which produces only small amounts of samples, the fundamentals developed by HPT should be used in the future to develop new methods with upscaled sample sizes and higher potential for industrial applications. This last issue is a general requirement of SPD for future commercialization in almost any application [173].

## 4. Conclusions

Global warming has become a significant concern in recent years, which seriously threatens the life of creatures. Conversion of CO_2_ molecules to other components, such as CO, is a way to stand this event. In this regard, photocatalytic CO_2_ conversion, which uses solar irradiation as a clean energy source, has been introduced as a new and promising strategy in recent years. Despite the introduction of various materials, which are modified by various strategies, the efficiency of CO_2_ photoreduction is still low compared with conventional methods for CO_2_ conversion. High-pressure torsion (HPT) as a severe plastic deformation (SPD) method has been used recently to produce some of the most active photocatalysts for CO_2_ conversion. The HPT method can increase the CO_2_ photoreduction efficiency by (i) oxygen vacancy and strain engineering, (ii) the stabilization of high-pressure phases, (iii) the formation of defective high-entropy oxides, and (iv) the synthesis of low-bandgap oxynitrides.

## Figures and Tables

**Table 1:** Summary of major publications about ceramics treated by high-pressure torsion and their major properties and applications.

Materials	Investigated Properties and Applications	Reference
Various Materials	Impact of pressure and strain on allotropy	Bridgman (1935) [56]
α-Al_2_O_3_	Microstructure and mechanical properties	Edalati et al. (2010) [57]
ZrO_2_	Allotropic phase transformations	Edalati et al. (2011) [58]
CuO	Dielectric properties	Makhnev et al. (2011) [59]
CuO, Y_3_Fe_5_O_12_, FeBO_3_	Optical properties and electronic structure	Gizhevskii et al. (2011) [60]
ZrO_2_	Phase transformation	Delogu et al. (2012) [61]
Cu_2_O, CuO	Middle infrared absorption and X-ray absorption	Mostovshchikova et al. (2012) [62]
CuO, Y_3_Fe_5_O_12_, FeBO_3_	Optical properties	Telegin et al. (2012) [63]
BaTiO_3_	Optical and dielectric properties	Edalati et al. (2015) [64]
TiO_2_-II	Photocatalytic activity for hydrogen production	Razavi-Khosroshahi et al. (2016) [23]
Various Materials	Review on HPT	Edalati et al. (2016) [40]
TiO_2_	Plastic strain and phase transformation	Razavi-Khosroshahi et al. (2016) [65]
Y_2_O_3_	Optical properties	Razavi-Khosroshahi et al. (2016) [66]
YBa_2_Cu_3_O_y_	Microstructural investigation	Kuznetsova et al. (2017) [67]
BN	Coupled elastoplasticity and plastic strain-induced phase transformation	Feng et al. (2017) [68]
ZnO	Photocatalytic activity for dye degradation	Razavi-Khosroshahi et al. (2017) [26]
Fe_3_O_4_	Lithium-ion batteries	Qian et al. (2018) [69]
ZnO	Plastic flow and microstructural instabilities	Qi et al. (2018) [70]
Fe_71.2_Cr_22.7_Mn_1.3_N_4.8_	Microstructural features	Shabashov et al. (2018) [71]
BN	Modeling of plastic flow and strain-induced phase transformation	Feng et al. (2019) [72]
TiO_2_-II	Electrocatalysis for hydrogen generation	Edalati et al. (2019) [73]
γ-Al_2_O_3_	Photocatalytic activity for dye degradation	Edalati et al. (2019) [27]
Various Oxides	Review on HPT of oxides	Edalati et al. (2019) [74]
MgO	Photocatalytic activity for dye degradation	Fujita et al. (2020) [28]
ZrO_2_	Photocatalytic activity for hydrogen production	Wang et al. (2020) [26]
SiO_2_	Photocatalytic activity for dye degradation	Wang et al. (2020) [34]
CsTaO_3_, LiTaO_3_	Photocatalytic activity for hydrogen production	Edalati et al. (2020) [24]
GaN-ZnO	Photocatalytic activity for hydrogen production	Edalati et al. (2020) [25]
Fe_53.3_Ni_26.5_B_20.2_, Co_28.2_Fe_38.9_Cr_15.4_Si_0.3_B_17.2_	Microstructure and mechanical properties	Permyakova et al. (2020) [75]
TiHfZrNbTaO_11_	Photocatalytic activity for hydrogen production	Edalati et al. (2020) [27]
TiO_2_-ZnO	Photocatalytic activity for hydrogen production	Hidalgo-Jimeneza et al. (2020) [28]
Bi_2_O_3_	Enhanced photocurrent generation	Fujita et al. (2020) [76]
TiO_2_-II	Visible-light photocurrent generation	Wang et al. (2020) [77]
TiO_2_-II	Photocatalytic activity for CO_2_ conversion	Akrami et al. (2021) [30]
TiZrHfNbTaO_6_N_3_	Photocatalytic activity for hydrogen production	Edalati et al. (2021) [29]
SiO_2_, VO_2_	Phase transformation	Edalati et al. (2021) [78]
TiO_2_	Grain coarsening and phase transformation	Edalati et al. (2021) [79]
ZnO	Bandgap narrowing	Qi et al. (2021) [80]
BiVO_4_	Photocatalytic activity for CO_2_ conversion	Akrami et al. (2022) [29]
TiHfZrNbTaO_11_	Photocatalytic activity for CO_2_ conversion	Akrami et al. (2022) [31]
TiZrNbTaWO_12_	Photocatalytic activity for oxygen production	Edalati et al. (2022) [30]
TiZrHfNbTaO_6_N_3_	Photocatalytic activity for CO_2_ conversion	Akrami et al. (2022) [32]

**Table 2:** Photocatalytic CO production rate on high-entropy oxynitride TiZrNbHfTaO_6_N_3_ synthesized by high-pressure torsion compared with photocatalysts reported in the literature.

Photocatalyst	Catalyst Concentration	Light Source	CO Production Rate (µmolh^−1^g^−1^)	CO Production Rate (µmolh^−1^m^−1^)	Ref.
TiO_2_/Graphitic Carbon	100 mg (Gas System)	300 W Xenon	10.16	0.04	Wang et al. (2013) [91]
Bicrystalline Anatase/Brookite TiO_2_ Microspheres	30 mg (Gas System)	150 W Solar Simulator	145	0.95	Liu et al. (2013) [92]
Ag/TaON/RuBLRu′	2 gL^−1^ (Liquid System)	500 W High-Pressure Mercury	0.056	----	Sekizawa et al. (2013) [93]
10 wt % Montmorillonite-Loaded TiO_2_	50 mg (Gas System)	500 W Mercury	103	1.25	Tahir et al. (2013) [94]
Anatase TiO_2_ Nanofibers	50 gL^−1^ (Liquid System)	500 W Mercury Flash	40	-----	Zhang et al. (2013) [95]
TiO_2_ Nanosheets Exposed {001} Facet	1 gL^−1^ (Liquid System)	Two 18 W Low-Pressure Mercury	0.12	0.00095	He et al. (2014) [96]
Anatase TiO_2_ Hierarchical Microspheres	200 mg (Gas System)	40 W Mercury UV	18.5	0.37	Fang et al. (2014) [97]
TiO_2_ and Zn(II) Porphyrin Mixed Phases	60 mg (Gas System)	300 W Xenon	8	0.062	Li et al. (2015) [98]
Anatase TiO_2_ Hollow Sphere	100 mg (Gas System)	40 W Mercury UV	14	0.16	Fang et al. (2015) [99]
10 wt % In-Doped Anatase TiO_2_	250 mg (Gas System)	500 W Mercury Flash	81	1.33	Tahir et al. (2015) [100]
Pt^2+^–Pt^0^/TiO_2_	100 mg (Gas System)	300 W Xenon	~12.14	0.7	Xiong et al. (2015) [101]
BiOI	150 mg (Gas System)	300 W High-Pressure Xenon	4.1	0.03	Ye et al. (2016) [102]
RuRu/Ag/TaON	1 gL^−1^ (Liquid System)	High-Pressure Mercury	5	----	Nakada et al. (2016) [103]
RuRu/TaON	1 gL^−1^ (Liquid System)	High-Pressure Mercury	3.33	----	Nakada et al. (2016) [103]
CeO_2-x_	50 mg (Gas System)	300 W Xenon	1.65	0.08	Ye et al. (2017) [104]
Cu_2_O/RuO_x_	500 mg (Gas System)	150 W Xenon	0.88	---	Pastor et al. (2017) [105]
TiO_2_ 3D Ordered Microporous/Pd	100 mg (Gas System)	300 W Xenon	3.9	0.066	Jiao et al. (2017) [106]
BiVO_4_/C/Cu_2_O	---	300 W Xenon	3.01	----	Kim et al. (2018) [107]
g-C_3_N_4_/α-Fe_2_O_3_	200 mg (Gas System)	300 W Xenon	5.7	-----	Wang et al. (2018) [108]
xCu_2_O/Zn_2-2x_Cr	4 gL^−1^ (Liquid System)	200 W Mercury-Xenon	2.5	0.018	Jiang et al. (2018) [109]
TiO_2_/Carbon Nitride Nanosheet	25 mg (Gas System)	150 W Xenon	2.04	----	Crake et al. (2019) [110]
TiO_2_/CoOx Hydrogenated	50 mg (Gas System)	150 W UV	1.24	0.0045	Li et al. (2019) [111]
Bi_4_O_5_Br_2_	20 mg (Gas System)	300 W High-Pressure Xenon	63.13	0.58	Bai et al. (2019) [112]
ZnGaON	---	1600 W Xenon	1.05	---	Maiti et al. (2019) [113]
C_3_N_4_ by Thermal Condensation	100 mg (Gas System)	350 W Mercury	4.83	------	Xia et al. (2019) [9]
Cd_1-x_Zn_x_S	45 mg (Gas System)	UV-LED Irradiation	2.9	0.015	Kozlova et al. (2019) [114]
Bi_24_O_31_C_l10_	50 mg (Gas System)	300 W High-Pressure Xenon	0.9	---	Jin et al. (2019) [115]
Bi_2_Sn_2_O_7_	0.4 gL^−1^ (Liquid System)	300 W Xenon	14.88	0.24	Guo et al. (2020) [116]
Ag/Bi/BiVO_4_	10 mg (Gas System)	300 W Xenon Illuminator	5.19	0.42	Duan et al. (2020) [117]
g-C_3_N_4_/BiOCl	20 mg (Gas System)	300 W High-Pressure Xenon	4.73	---	Chen et al. (2020) [118]
Fe/g-C_3_N_4_	1 gL^−1^ (Liquid System)	300 W Xenon	~22.5	0.06	Dao et al. (2020) [119]
Bi_2_MoO_6_	0.7 gL^−1^ (Liquid System)	300 W Xenon	41.5	1.26	Zhang et al. (2020) [120]
g-C_3_N_4_/Zinc Carbodiimide/Zeolitic Imidazolate Framework	100 mg (Gas System)	300 W Xenon	~0.45	0.014	Xie et al. (2020) [121]
WO_3_/LaTiO_2_N	10 mg (Gas System)	300 W Xenon	2.21	0.4	Lin et al. (2021) [122]
α-Fe_2_O_3_/LaTiO_2_N	20 mg (Gas System)	300 W Xenon	9.7	0.65	Song et al. (2021) [123]
TiZrHfNbTaO_6_N_3_	0.2 gL^−1^ (Liquid System)	400 W High-Pressure Mercury	10.72 ± 1.77	4.66 ± 0.3	Akrami et al. (2022) [32]

## Data Availability

No new data were created or analyzed in this study. Data sharing is not applicable to this article.
